# Feline and Canine Coronaviruses: Common Genetic and Pathobiological Features

**DOI:** 10.1155/2011/609465

**Published:** 2011-07-31

**Authors:** Sophie Le Poder

**Affiliations:** UMR 1161 INRA-ENVA-ANSES, 7 avenue Général de Gaulle, 94704 Maisons-Alfort, France

## Abstract

A new human coronavirus responsible for severe acute respiratory syndrome (SARS) was identified in 2003, which raised concern about coronaviruses as agents of serious infectious disease. Nevertheless, coronaviruses have been known for about 50 years to be major agents of respiratory, enteric, or systemic infections of domestic and companion animals. Feline and canine coronaviruses are widespread among dog and cat populations, sometimes leading to the fatal diseases known as feline infectious peritonitis (FIP) and pantropic canine coronavirus infection in cats and dogs, respectively. In this paper, different aspects of the genetics, host cell tropism, and pathogenesis of the feline and canine coronaviruses (FCoV and CCoV) will be discussed, with a view to illustrating how study of FCoVs and CCoVs can improve our general understanding of the pathobiology of coronaviruses.

## 1. Introduction

Coronaviruses are enveloped viruses with a large (27–32 kb) single-stranded, positive-sense RNA [1]. The genome includes at least 6 open reading frames (ORFs) flanked by 5′ and 3′ untranslated regions. The viral RNA is packaged by the nucleocapsid protein (N), which are themselves enclosed in an envelope containing at least three virally-encoded membrane proteins: the spike (S) glycoprotein, transmembrane protein (M), and small membrane protein (E) [2, 3]. Some coronaviruses have an additional membrane glycoprotein, hemagglutinin esterase [4].

The trimeric S protein forms characteristic viral peplomers that are involved in virus attachment to cell receptors and in virus-cell fusion [5, 6]. The M protein, the most abundant structural component, is a type III glycoprotein consisting of a short amino-terminal ectodomain, a triple-spanning transmembrane domain, and a carboxyl-terminal inner domain [7]. The E protein has been found to be important for viral envelope assembly [8]. 

 Coronaviruses infect many animals species, including cats and dogs. Feline infectious peritonitis (FIP) was first recognized in 1963 at the Angell Memorial Animal Hospital in Boston by Holzworth [9]. A few years later, Ward discovered that the etiologic agent of this disease was a virus of the family* Coronaviridae*, that is, the feline coronavirus (FCoV) [10]. The first observation of canine coronavirus (CCoV) infection was reported in 1971, when Binn and colleagues isolated a coronavirus (strain 1-71) from dogs with acute enteritis in a military canine unit in Germany [11]. Since these discoveries, much knowledge has been gained as regarding the molecular biology and pathobiology of these viruses. This paper describes recent advances in knowledge of their genetic diversity, the determinants of pathogenesis, and their ability to cross the species barrier. Differences and similarities between these viruses have been highlighted. The paper focuses on feline and canine coronaviruses of the *Alphacoronavirus* genus, and leaves the canine respiratory coronavirus, which belongs to the *Betacoronavirus* genus, aside (see below).

## 2. Classification of Feline and Canine Coronaviruses

### 2.1. Taxonomy

The family *Coronaviridae* now comprises two subfamilies, *Coronavirinae* and *Torovirinae*, which display similarities in morphology, genomic organization, and gene expression [12, 13]. On the basis of genetic and serological properties, the *Coronavirinae *subfamily has been divided into three new genera, *Alpha*-, *Beta-*, and *Gammacoronavirus *(formerly named group 1, 2 and 3, resp.) [14]. Each genus is subdivided into different species on the basis of sequence identity in the replicase domains of the polyprotein pp1ab. Representative members of each species are listed in Table 1. The porcine transmissible gastroenteritis virus (TGEV), canine coronavirus (CCoV), and feline coronavirus (FCoV) display greater than 96% sequence identity within the replicase polyprotein pp1ab, and for this reason have been grouped in the same species, *alphacoronavirus 1*, within the* Alphacoronavirus *genus [14] (Table 1). Both the ferret enteric coronavirus (FRECV) and the ferret systemic coronavirus (FRSCV) also belong to the *Alphacoronavirus *genus [15]. Recently, the genome of the mink coronavirus (MCoV), the etiological agent of catarrhal gastoenteritis in mink, has been completely sequenced and shows close genetic relationship with FRECV and FRSCV. The authors proposed to group the ferret and mink coronaviruses in a new *alphacoronavirus* species (*alphacoronavirus 2*) within the *Alphacoronavirus* genus [16].

### 2.2. FCoV and CCoV Genotypes

**Figure 1:**
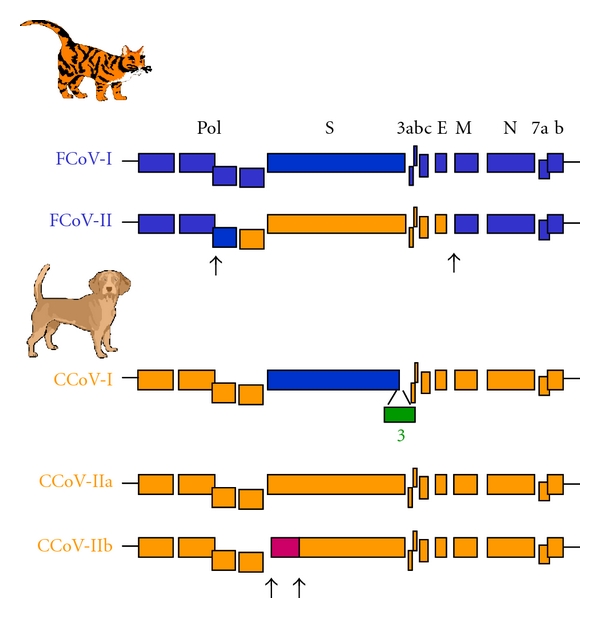
Genetic relationships between the different feline and canine coronaviruses genotypes (FCoV and CCoV). The feline sequences are coloured in blue, the canine sequences in orange, and the porcine sequences in purple. Arrows indicate the putative sites of recombinations. The genes encoding for the polymerase polyprotein (pol), the structural spike (S), the envelope (E), the membrane (M), and the nucleocapsid (N) proteins are indicated. The genes encoding the accessory proteins are designated by numerals.

FCoV and CCoV strains are classified into 2 main genotypes, which are schematized in Figure 1, in which their phylogenetic relationships are highlighted.

Historically, the two FCoV genotypes have been distinguished by in vitro virus neutralization assays, using either type-specific feline sera or monoclonal antibodies raised against the S protein [17, 18]. Advances in genetic analyses have revealed that type II FCoVs originate from a double recombination between type I FCoV and CCoV, resulting in a genome principally composed of FCoV sequences but with the S gene and its adjacent sequences originating from CCoV [19–22]. FCoV is highly prevalent in catteries, where up to 80% of the animals are seropositive, while in households 10 to 50% of the cats are infected [23]. In the field, the FCoV serotype I is preponderant, causing 80% to 95% of infections [24–27]. However, most research studies have been conducted with type II, which can be easily propagated in cell cultures.

Our knowledge of the molecular biology of CCoV has accumulated rapidly since the early 2000s. Genetic analysis of several CCoVs circulating in Italy first revealed a new canine genetic cluster bearing point mutations within the M gene that increased similarity to the feline homolog [28]. The new genotype was initially designated “FCoV-like CCoVs”. Further, the S sequence analyse showed that these strains segregated with FCoV-I (about81% identity) rather than with the reference CCoVs (about54% identity) [29]. Finally, on the basis of their geneticrelation to FCoV-I, FCoV-like CCoVs were designated as CCoV type I and the typical reference CCoVs have been called CCoV type II [30]. Unlike FCoVs, the two CCoV genotypes are commonly detected simultaneously in the same dog, thus allowing genetic recombination to occur [31, 32]. Recently, an additional ORF, named ORF3, located between the end of the S gene and the ORF3a gene, was discovered in CCoV-I strains. This gene is absent in all other alphacoronaviruses studied so far (Figure 1). ORF3 encodes a 28 kDa N-glycosylated protein with a cleavable N-terminal signal, the function of which is unknown [33]. These data provide insight into the evolutionary history of FCoV and CCoV. It has been proposed that type I FCoV and CCoV originated from a common ancestor. CCoV-I may have acquired the ORF3 gene after the divergence of FCoV-I, or, alternatively, FCoV-I may have lost the ORF3 gene present in their common ancestor. The acquisition of a new S gene led to the emergence of CCoV-II, which in turn gave rise to FCoV-II through recombination with FCoV-I [33].

CCoV and TGEV also appear to be closely linked. TGEV probably originated from CCoV-II [33, 34]. Subsequent recombination between these viruses led to the emergence of a new CCoV-II cluster (formerly named TGEV-like CCoVs), in which the N-terminus of the spike protein was highly similar to TGEV whereas the rest of the genome clustered with reference CCoV-II isolates (Figure 1) [35]. Taking into account this discovery, the CCoV-II genotype has been subdivided into two different subtypes, CCoV-IIa and CCoV-IIb, comprising reference and TGEV recombinant isolates, respectively. CCoV-IIb was first identified in Italy and in the United Kingdom [35, 36]. A recent study was conducted to establish the prevalence of the various canine genotypes in Europe. It appeared that CCoV-I accounts for about 20% of the CCoV infections, and CCoV-II for 44%, with nearly 36% of infected dogs being coinfected by both genotypes. Moreover, the CCoV-IIb subtype was detected in 20% of the CCoV-II infections [37].

### 2.3. FCoV and CCoV Biotypes

 For many years, FCoVs have been classified into different biotypes on the basis of their pathobiology. Avirulent strains, which usually induce mild or subclinical symptoms, are referred to as feline enteric coronavirus (FECV) [38]. Virulent strains cause feline infectious peritonitis and are called feline infectious peritonitis viruses (FIPV). Until 2005, CCoVs were considered to be mild enteropathogens. In 2005, a virulent variant causing systemic disease in pups and mortality was first recognized in Italy [39]. This virulent biotype has been named canine pantropic coronavirus in reference to its systemic distribution in internal organs [39, 40]. Interestingly, ferret coronaviruses are also classified according to their virulence. The ferret enteric coronavirus (FRECV), which is widely distributed, causes an enteric disease called epizootic catarrhal enteritis, whose overall mortality rate is low [41]. By contrast, the highly pathogenic ferret systemic coronavirus (FRSCV) induces FIP-like disease [42, 43].

Both feline genotypes may be responsible for mild enteric or FIP diseases. FIP remains a rare event, and only a minority of FCoV-infected cats (up to 10%) develop the illness [24, 44]. Two forms of FIP are recognized: the wet/effusive form with accumulation of a characteristic viscous yellow fluid in body cavities and the dry/noneffusive form with pyogranulomatous lesions affecting several organs [45]. Both forms are progressive and ultimately fatal [46]. FIP is often observed in young cats [47, 48]. In ferrets infected with FRSCV, the gross lesions resemble those described in cats with the dry form of FIP. Again, histologic lesions are characterized by severe pyogranulomas commonly observed in the mesentery and the peritoneal surface [42].

Both canine genotypes have been associated with enteric CCoV. By contrast, pantropic CCoVs identified so far all belong to the CCoV-IIa genetic cluster [49]. Enteric CCoV infection does not prevent subsequent infection with the pantropic variant [50]. Dogs seropositive for enteric CCoVs are still susceptible to pantropic viruses, but the clinical signs are moderate by comparison with those in seronegative dogs, probably owing to partial cross-protection induced by antibodies against enteric CCoV [50]. During infection with the enteric CCoV, the virus remains restricted to the gastrointestinal tract. Conversely, the highly virulent pantropic CCoV is detected at high titres in lungs, spleen, liver, kidney, and brain [39]. Clinical signs consist of fever, lethargy, haemorrhagic diarrhoea, severe lymphopenia, and neurological signs followed by death [39, 49]. The prevalence of the canine pantropic coronavirus is yet unknown, and further epidemiological studies are required to determine its distribution in dog populations. A pantropic strain (CB/05) has been successfully isolated from the lungs of a dead pup. CB/05 has subsequently been used to reproduce the disease experimentally, thereby improving understanding of this new illness. Infection with the CB/05 strain has demonstrated that disease outcome depends on the age at infection. Puppies over 6 months old may recover, whereas younger puppies (2-3 months) develop the most severe symptoms [51]. Lymphopenia is one of the main features of pantropic CCoV infection under natural and experimental infections. While a transient reduction in T and B cell populations is observed during the first week after infection, the CD4^+^ T cell population remains depleted for 30 days postinfection, which could cause dysfunction of the immune system and favour opportunistic infections [52].

## 3. Viral Life Cycle

### 3.1. Target Cells

The cell tropism of FCoVs and CCoVs has been studied by experiments conducted in host species. In the case of FIP, the virus mainly infects cells of the monocyte/macrophage lineage. Circulating FIPV-infected monocytes are thought to disseminate the virus to many organs [45]. Conversely, in asymptomatic cats, FECV is mainly confined to the intestine and presumed to replicate in enterocytes. It is believed that the capacity of an FCoV strain to replicate in monocyte-macrophage cells is correlated with its virulence [53]. Experiments in which FIPVs and FECVs were compared for their ability to replicate in isolated peripheral blood monocytes or in peritoneal macrophages have corroborated this assumption [53–55]. Monocytes and macrophages are less likely to support FECV infections. Furthermore, the viral cycle is less productive than with FIPV. In comparison, no differences were noted when the same experiments were conducted on Crandell feline kidney (CrFK) cells [55]. 

The life cycle of canine coronavirus has been essentially studied in the canine fibrosarcoma cell line (A-72 cells). Infection led to apoptosis, which may be responsible for pathology induced by CCoV infection [56, 57]. Like FECV, enteric CCoV is thought to infect enterocytes, whereas the cell tropism of pantropic coronavirus remains unclear. Immunohistochemistry performed on tissues recovered from infected dogs led to the detection of coronavirus antigens in macrophages from different organs, reminiscent of FIP infection of cats [40]. Blood monocytes may also support viral replication, as suggested by the presence of viral RNA in blood leucocytes [58]. At present, experiments conducted in isolated blood monocytes or bone marrow-derived macrophages, such as those described for FIPV, have not been performed. Such assays may be necessary in the future to determine the importance of macrophages in the pathobiology of the pantropic coronavirus. Finally, the infection of immature lymphocytes cannot be excluded since high levels of RNA were found in the thymus, possibly explaining the severe depletion in the CD4^+^ cell population [52].

Interestingly, the target cells of ferret coronaviruses are probably the same as those of FCoVs and CCoVs. Again, enterocytes are susceptible to the mild FRECV, whereas macrophages seem to have a pivotal role in the pathobiogenesis of the virulent ferret systemic coronavirus [43, 59, 60].

### 3.2. Attachment and Entry

Like other CoVs, FCoVs and CCoVs require the viral S protein, a class 1 fusion protein, for cell entry. Attachment to the cellular receptor is mediated by the N-terminal domain of the S protein [61], while fusion of the viral envelope with host cell membranes is mediated by the C-terminal domain [62]. Like most class 1 fusion proteins, the S protein of CoVs of the *beta*- and *gammacoronavirus* genera harbours a cleavage site between the S1 and S2 domains [63]. Only recently a similar furin cleavage motif (RRXRR) has been recognized in the FCoV strains UCD and UCD8 and in the CCoV-I strain Elmo/02, approximately in the same position as in *beta*- and *gammacoronaviruses *[29, 64]. 

The cellular receptor identified for the *alphacoronavirus 1* species is the aminopeptidase N protein (APN or CD13) [65]. APN is a 150–160 kDa type II glycoprotein and a metalloprotease. APN is expressed on the cell surface of epithelial cells of the kidney, intestine, and respiratory tract, and in granulocytes, monocytes, fibroblasts, endothelial cells, cerebral pericytes at the blood-brain barrier, and synaptic membranes in the CNS [66–68]. APN certainly serves as a receptor for FCoV-II and CCoV-II, but probably not for FCoV-I and CCoV-I. Using mouse monoclonal antibodies as blocking agents, Hohdatsu et al. noted differences in receptors for type I and II FCoVs and suggested that feline APN is a receptor only for type II FCoVs [69]. More recently, Dye et al. produced retroviral pseudotypes that bear the S glycoprotein of type I or type II FCoV and demonstrated that feline APN is not used by type I FCoV S glycoprotein for the virus entry [70]. Finally, with chimeric viruses carrying either a type I or a type II spike, Tekes et al. confirmed that feline APN is not the functional receptor of FCoV-I [71]. Considering the strong similarity between the spikes of CCoV-I and FCoV-I, it is tempting to speculate that CCoV-I uses the same unknown receptor as FCoV-I. Certain coronaviruses also use a variety of coreceptors during entry, including C-type lectins; indeed L-SIGN facilitates the infection of both SARS-CoV and HCoV-229E [72, 73]. Regan et al. showed that type-I and -II FCoVs can use DC-SIGN as a co-receptor for cellular entry [74]. DC-SIGN is considered to be widely expressed in monocyte-derived macrophages, which are thought to be the targets of FIPV infection in vivo [75]. It seems, however, that the FIPV and FECV biotypes used DC-SIGN in a similar manner, which suggests that the difference in cell tropism between these viruses does not depend on the use of DC-SIGN. The role of lectins in the entry of CCoVs has not been studied so far.

Coronaviruses enter cells via endocytosis and not via direct fusion of the viral envelope with the plasma membrane [76–78]. It has been shown that HCoV-229E binds human APN in rafts and enters human fibroblasts through caveolae [77]. As regarding entry of FCoV-II, studies have been conducted with the 79-1146 strain, which belongs to the FIPV biotype. In monocytes, this virus is internalized through a novel clathrin- and caveolae-independent pathway that depends essentially on dynamin [79]. This is the first report of an internalization pathway with these properties and further investigation is required to determine whether use of this pathway is peculiar to the FIPV biotype. At present, no data have been published concerning the entry mechanism of CCoVs.

### 3.3. Replication-Transcription

With most studies focusing on TGEV or murine hepatitis virus (MHV), gene expression and replication of FCoV or CCoV have not been specifically studied as yet. The replicase-transcriptase proteins are encoded by ORF1a and ORF1b and are initially synthesized as two large polyproteins, pp1a and pp1ab. These polyproteins are processed by two or three viral proteases to generate 16 end products, termed nsp1 to nsp16 [13, 80]. These cleavage products assemble into the replication-transcription complex, which promotes genome replication and subgenomic mRNA synthesis [81]. The methyltransferase activity of nsp16 has been extensively studied by using recombinant FCoV nsp16. Decroly et al. provide experimental evidence that FCoV nsp16 specifically binds capped RNAs of 3 to 6 nucleotides in length carrying a methyl group at the N7 position of the guanosine cap, referred as cap-0 structure. Nsp16 methylates the ribose of the first nucleotide of the RNA and participates in the conversion of viral RNAs from a cap-0 to a cap-1 structure [82]. This function is probably common to all coronaviruses.

Like all CoVs, the transcription of FCoVs and CCoVs is characterized by the production of multiple subgenomic mRNAs that contain sequences corresponding to both ends of the genome. The generation of subgenomic mRNAs involves a process of discontinuous transcription, by mechanisms that have principally been studied in TGEV [83].

## 4. Role of FCoV and CCoV Proteins in Pathogenesis

The molecular determinants that may account for the dramatic difference in pathogenesis between FECV and FIPV have been extensively investigated. Today, FIPV is considered to be a genetic variant of enteric FECV and I shall focus in this chapter on the mutations probably implicated in virulence. It is likely, however, that host immunity also plays a role in the development of FIP. The pantropic CCoV has been described only recently, and there is little information about the molecular determinants its increased virulence.

### 4.1. Role of the Spike Protein

Investigation of recombinant coronaviruses, including MHV, TGEV, and IBV, has conclusively demonstrated that the spike is an essential determinant for the pathogenicity of these viruses [84–86]. As regarding FIPV, the spike protein has been identified as critical for efficient macrophage infection. A chimeric virus in which the S protein of FECV strain 79-1683 replaces that of FIPV 79-1146 poorly infects macrophages, whereas the high virulent FIPV 79-1146 replicates efficiently in this cell type. Interestingly, the determinant of macrophage tropism was not localized within the receptor binding domain of the spike, but rather in the C-terminal domain responsible for membrane fusion [54]. This study, however, was based on laboratory strains of FCoV-II, which harbour an S gene arising from CCoV. More data for FCoV-I strains are necessary to confirm this hypothesis. 

To date, only one pantropic CCoV strain (CB/05) has been sequenced and compared to avirulent CCoVs. Curiously, the S protein displayed the highest degree of identity to FCoV-II strain 79-1683. Only residues Pro-73, Asn-125, and Ala-407 were peculiar to strain CB/05. A substitution at position 125 (Asp to His instead of to Asn) was also found in the BGF10 strain, a hypervirulent enteric strain [87].

### 4.2. Role of the Membrane Protein

Aside from its role in viral assembly, the coronavirus M protein is believed to be involved in host interactions. Regarding TGEV, the M protein has been shown to have interferogenic activity [88]. By comparison of viral sequences from 48 healthy and 8 FIP-infected cats, Brown et al. discovered 5 amino acid differences located in the transmembrane domain and in the cytoplasmic tail of the membrane protein. The authors suggested that these findings could be used as diagnostic markers for FIPV, which would represent a significant advance in management of FIP [89]. This is the first study suggesting a role for the M protein in FIPV pathogenesis, and sequences from additional cats with FIP are required to draw definitive conclusions.

### 4.3. Role of the Nucleocapsid Protein

The N protein, which is necessary for virus assembly, is involved in the formation of the transcription complex and in pathogenesis, at least for MHV [90]. The N protein from MHV stimulates the expression of a gene implicated in the development of fulminant hepatitis [91, 92]. Two phylogenetic studies have been conducted on FIPV- and FECV-infected cats. Both revealed genetic and antigenic variation of N, but without relation to the FIPV and FECV biotypes [93, 94].

### 4.4. Role of Accessory Proteins

Coronaviruses encode small nonstructural proteins of unknown function, which are specific to each genus of coronaviruses. The genome of FCoVs and CCoVs includes two gene clusters encoding nonstructural proteins: the ORFs 3a, 3b, 3c (located between the S and E genes) and ORFs 7a, 7b (located downstream of the N gene). Evidence for their role in the FIPV pathotype comes from sequence comparisons of FECV and FIPV field strains and from reverse-genetic experiments. Haijema et al. deleted the gene clusters ORF 3abc or 7ab from the highly virulent FIPV strain 79-1146 and obtained deletion mutant viruses that multiplied efficiently in cell culture but that were attenuated in cats [95]. Epidemiological studies have also corroborated the importance of these genes. FIPV strains frequently (up to 70% depending on the study) carry mutations that specifically inactivate ORF 3c, whilst FECVs possess a fully functional 3c gene [96–98]. An intact 3c gene is apparently essential for efficient replication in the intestinal tract. ORF 3c encodes an accessory triple-spanning membrane protein, 238 residues in length. Its predicted topology is similar to that of the M protein and the 3a protein of SARS-CoV, despite their high degree of sequence diversity [99]. Since some FIPVs appear to have intact 3c genes, it is likely that alternative mutations can generate the virulent FIPV biotype.

ORF 7b is specific to FCoVs, CCoVs, and ferret coronaviruses [15, 100]. It encodes a soluble nonstructural glycoprotein of 24 kDa, whose function remains enigmatic [101]. Like ORF 3c, its expression is not indispensable for in vitro replication. Conversely, in almost all natural FECV infections, ORF 7b is maintained and the appearance of an FIPV biotype often correlates with the loss of ORF 7b expression [89, 98]. Nevertheless, another study has suggested that 7b deletion occurs in both FIPV and FECV infections [102]. Altogether, the switch from FECV to FIPV could be a multistep process, involving mutations in at least the S and accessory genes. Complete sequences of FECV and FIPV field strains will be necessary to validate this assumption.

Regarding the pantropic CCoV, the most striking genetic marker identified in the unique pantropic CCoV genome sequenced (strain CB/05) consisted of a 38-nt deletion in ORF3b, which is predicted to give rise to a truncated nonstructural protein 3b [39, 49]. This observation needs to be confirmed by analysis of additional pantropic sequences.

## 5. Interspecies Transmission

Coronaviruses are characterized by a significant capacity for genetic change that enables them to adapt to new hosts and ecological niches, sometimes causing zoonotic outbreaks with disastrous consequences like the SARS epidemic in 2003 [103]. In this chapter, I shall discuss the possibility of heterospecific coronavirus infections in cats or dogs. 

### 5.1. The Specific Properties of Feline APN

In general, the APN receptor is used by alphacoronaviruses in a species-specific manner, that is, human APN is the cellular receptor for HCoV-229E, but not for the porcine coronaviruses, and conversely, porcine APN serves as a receptor for the porcine coronaviruses, but not for HCoV-229, FCoV, or CCoV [104, 105]. However, feline APN is a functional receptor for many alphacoronaviruses, including feline (FECV and FIPV), human (HCoV-229E), porcine (TGEV), and canine coronaviruses [106]. Human, feline, and porcine APN show strong amino acid conservation and display about 78% identity. Yet, species-specific tropism is influenced by minor differences in certain regions of APN [107]. Chimeras of mouse-feline APN were used by Tusell et al. to identify the three small, discontinuous regions in feline APN that are critical determinants for the host range of these coronaviruses. Amino acids (aa) 288 to 290 are essential for the entry of HCoV-229E, particularly the presence of an N-glycosylation sequon prevents virus infection. TGEV requires the region corresponding to aa 732 to 746 of feline APN, while FCoV and CCoV necessitate both aa 732 to 746 and aa 764 to 788 for entry [108]. The entry of all of these viruses is blocked by the same monoclonal antibody directed against feline APN, suggesting that these three regions are closely link together in the three dimensional structure of feline APN. HCoV-229E, FCoV, TGEV, and CCoV probably evolved from the same ancestral alphacoronavirus, which may have infected cats using feline APN. The selection of mutations in the S protein may then have led to the appearance of viruses able to infect other host species by means of their cognate APN proteins, although all of them retained their capacity to use feline APN as a receptor in vitro.

### 5.2. Cross-Species Jump between Cats and Dogs?

Considering the exceptional properties of feline APN, cats could be infected by HCoV-229E, TGEV, or CCoV. In vivo, under experimental conditions, cats can be infected with CCoV and with human HCoV-229E without developing symptoms [109–111]. However, nonfeline coronaviruses have never been formally reported in naturally infected cats. Considering the close genetic relationship between feline and canine coronaviruses, interspecific circulation of either CCoV in cats or FCoV in dogs is plausible. The genomic organisation of FCoV-II strongly suggests that coinfection with FCoV-I and CCoV-II occurred in one of these species, which led, after a double recombination event, to the emergence of FCoV-II. Moreover, FCoV-I/CCoV-I and FCoV-II/CCoV-II have a highly similar spike, which is a crucial determinant of the host species. In 2006, a study performed in an Austrian shelter and based on phylogenetic analysis of a fragment of the M gene did indeed suggest that some cats were infected with CCoV-I [112]. However, since the ORF3 gene had not been described at this time, it was impossible to confirm that these atypical strains belonged to the CCoV-I genotype.

Beyond the alphacoronaviruses, cats are also susceptible to SARS-CoV replication. After intratracheal inoculation, infected animals shed the virus from the pharynx from 2 to 10 days postinfection and transmitted the virus to animals with which they were in close contact [113]. Although none of the infected cats developed any symptoms, mild pulmonary histologic lesions were observed in these animals.

Experiments in which coronaviruses other than CCoVs have been administered to dogs have never been performed, and in the field only CCoVs sequences have been recovered from infected animals. However, sequence comparisons suggest that TGEV resulted from a cross-species jump of CCoV-II from dogs to pigs [34]. Furthermore, CCoV-IIb, only recently described, results from a double recombination between CCoV-II and TGEV, suggesting that coinfection has occurred in at least one host species [18].

## 6. Conclusion

Coronaviruses display unique molecular mechanisms of transcription and recombination. One of the most important insights gained over the past several years is that coronaviruses have crossed and in all likelihood will continue to cross between species, thus causing emerging disease in new host species, as was the case with the SARS epidemic in 2003. Coronaviruses of companion animal species were described long before the emergence of SARS-CoV. They exemplify the distinctive features of coronaviruses; that is, the presence of different biotypes and genotypes within each species, the critical role of accessory proteins in virulence and the possibility of interspecies transmission. FCoVs and CCoVs are common pathogens and readily evolve. It is necessary to pursue epidemiological surveillance of these viruses, so as to detect the emergence of new variants, which may have increased pathogenicity and/or a new host range, as early as possible. The knowledge accumulated about FCoVs and CCoVs, summarized in this paper, has made a substantial contribution to the understanding of the genetic evolution and pathobiology of coronaviruses. Observations that the spike protein and the accessory proteins contribute to pathogenesis and to host range have greatly benefited molecular investigation of the SARS-CoV. The next major goal will be to define the molecular determinants of virulence and tropism. Progress in these fields will require a better comprehension of the interactions between viral and host proteins and to what extent they are coronavirus- and organ-specific. In this context, study of FCoVs and CCoVs, as representative members of the *Coronaviridae* family, will again be helpful.

## Figures and Tables

**Table 1:** Coronavirus genera, species, and representative members.

Genus	Species	Acronym	Host
	*Alphacoronavirus 1*		
	Transmissible gastroenteritis virus	TGEV	Pig
	Feline enteric coronavirus	FECV	Feline
	Feline infectious peritonitis virus	FIPV	Feline
	Canine coronavirus	CCoV	Canine
	*Alphacoronavirus 2* ^a^		
	Ferret enteric coronavirus	FRECV	Ferret
	Ferret systemic coronavirus	FRSCV	Ferret
	Mink coronavirus	MCoV	Mink
*Alphacoronavirus*	*Human coronavirus 229E*	HCoV-229E	Human
	*Human coronavirus NL63*	HCoV-NL63	Human
	*Porcine epidemic diarrhea virus*	PEDV	Pig
	*Rhinolophus bat coronavirus HKU2*	Rh-BatCoV HKU2	Bat
	*Scotophilus bat coronavirus 512/05*	Sc-BatCoV 512	Bat
	*Miniopterus bat coronavirus 1*	Mi-BatCoV 1	Bat
	*Miniopterus bat coronavirus HKU8*	Mi-BatCoV HKU8	Bat

	*Betacoronavirus 1*		
	Human coronavirus OC43	HCoV-OC43	Human
	Bovine coronavirus	BCoV	Bovine
	Canine respiratory coronavirus	CRCoV	Canine
	Equine coronavirus	ECoV	Horse
	Porcine hemagglutinating encephalomyelitis virus	PHEV	Pig
	*Murine coronavirus*		
	Murine hepatitis virus	MHV	Mouse
	Rat sialodacryoadenitis virus	SDAV	Rat
*Betacoronavirus*	*Severe acute respiratory syndrome related coronavirus*		
	Severe acute respiratory syndrome coronavirus	SARS-CoV	Human
	SARS related Rhinolophus bat coronavirus	SARSr-Rh-BatCoV	Bat
	*Human coronavirus HKU1*	HCoV HKU1	Human
	*Rousettus bat coronavirus HKU9*	Ro-BaCoV HKU9	Bat
	*Tylonycteris bat coronavirus HKU4*	Ty-BatCoV HKU4	Bat
	*Pipistrellus bat coronavirus HKU5*	Pi-BatCoV HKU5	Bat

	*Avian coronavirus*		
	Infectious bronchitis virus	IBV	Chicken
*Gammacoronavirus*	Turkey coronavirus	TuCoV	Turkey
	*Beluga whale coronavirus SW1*	BWCoV SW1	Beluga whale

^a^Proposed as species by Vlasova et al. [16].
